# Captive and urban environments are associated with distinct gut microbiota in deer mice (*Peromyscus maniculatus*)

**DOI:** 10.1098/rsbl.2022.0547

**Published:** 2023-03-08

**Authors:** Jessica Diaz, Kent H. Redford, Aspen T. Reese

**Affiliations:** ^1^ Department of Ecology, Behavior, and Evolution, University of California, San Diego, La Jolla, CA 92093, USA; ^2^ Center for Microbiome Innovation, University of California, San Diego, La Jolla, CA 92093, USA; ^3^ Archipelago Consulting, Portland, ME 04112, USA; ^4^ School of Marine and Environmental Programs, University of New England, Biddeford, ME 2350, USA

**Keywords:** microbiome, captivity, urbanization, gut, *Peromyscus maniculatus*

## Abstract

Animals in captive and urban environments encounter evolutionarily novel conditions shaped by humans, such as altered diets, exposure to human-associated bacteria, and, potentially, medical interventions. Captive and urban environments have been demonstrated to affect gut microbial composition and diversity independently but have not yet been studied together. By sequencing the gut microbiota of deer mice living in laboratory, zoo, urban and natural settings, we sought to identify (i) whether captive deer mouse gut microbiota have similar composition regardless of husbandry conditions and (ii) whether captive and urban deer mice have similar gut microbial composition. We found that the gut microbiota of captive deer mice were distinct from those of free-living deer mice, indicating captivity has a consistent effect on the deer mouse microbiota regardless of location, lineage or husbandry conditions for a population. Additionally, the gut microbial composition, diversity and bacterial load of free-living urban mice were distinct from those of all other environment types. Together, these results indicate that gut microbiota associated with captivity and urbanization are likely not a shared response to increased exposure to humans but rather are shaped by environmental features intrinsic to captive and urban conditions.

## 1. Introduction

It is well established that environmental factors such as diet, geographical location and social interactions can impact the gut microbiota (e.g. [1–3]). It is therefore unsurprising that numerous studies show clear compositional differences between the gut microbiota of animals living in different environment types. For example, animals in their natural habitats (here ‘undeveloped’ environments) often harbour distinct microbiota from those ‘developed’ settings such as cities (e.g. [4–6]), zoos (e.g. [7–9]) or laboratories (e.g. [10,11]).

Previous research has compared the gut microbiota of animals from an undeveloped environment to those from a developed environment, yet no studies have directly compared multiple types of developed environments. Such a comparison is required to better understand how the gut microbiota differs between and within environment types and to contextualize previously observed patterns. For example, while it is known that city [4] or laboratory [10] populations have distinct microbiota from animals in undeveloped environments, it is still unclear how similar urban and laboratory microbiota are to each other and whether the same environmental forces drive microbial divergences from the undeveloped state.

Here, we analysed the gut microbial composition of deer mice (*Peromyscus maniculatus*) from six populations spanning four environment types: free-living undeveloped, free-living urban, captive zoo and captive laboratory. Deer mice are a meaningful study species for this particular comparison because they exhibit broad ecological flexibility with a range that covers much of North America [12]. Much of this range consists of undeveloped environments, although they can also be found living commensally in and around human settlements. Deer mice are also an increasingly valuable model species for biological research [13], including studies of the microbiome [14]. Through our multi-environment comparison of this ubiquitous rodent species, we sought to examine broad ecological patterns in microbial composition and help contextualize previous studies of captive and urban gut microbiota.

## 2. Methods

### (a) Subject populations and sample collection

A total of 99 faecal or intestinal samples from deer mice (*Peromyscus maniculatus*) were collected between 2018 and 2021 for this and other research projects. Adult deer mice were sampled in six locations spanning four environment types, two each for free-living and captive conditions (table 1; electronic supplementary material, figure S1). These samples were donated by collaborators, causing slight variations in collection procedure between populations. Some samples were collected as fresh faecal samples and others were dissected from the large intestine postmortem. However, the effect of sample type on microbial composition was minimal compared to our main variables of interest (i.e. environment type and condition; table 2), so this discrepancy is not expected to confound our main findings. Greater detail regarding collection procedures for each population can be found in the electronic supplemental material.

**Table 1.** Sample information.

environment type	location ID	condition	number of samples	sample type	collection site	collection year and season	sex	subspecies	diet
undeveloped	UN1	free-living	11	intestinal	Wallace Woods, PA	summer 2018	4F, 2 M, 5 unknown	unknown	wild (omnivorous, likely insects, seeds, grains, etc.)
	UN2	free-living	7	faecal	Yancey County, NC	spring-summer 2021	unknown	unknown	wild (omnivorous, likely insects, seeds, grains, etc.)
urban	FU	free-living	10	intestinal	Bronx, NY	summer 2020	2F, 1 M, 7 unknown	unknown	unknown, likely found materials in and around zoo
zoo	CZ	captive	29	faecal	Bronx, NY	summer 2020	unknown	unknown	mixture of rodent chow and fresh vegetables
laboratory	CL1	captive	22	faecal	Cambridge, MA	winter 2020	11F, 11M	11 *bairdii*, 11 *gambelii*	irradiated Prolab Isopro RMH 3000 + sunflower seeds
	CL2	captive	20	faecal	Columbia, SC	winter 2021	unknown	*bairdii*	irradiated Harlan 8904 Teklad Rodent Diet

**Table 2.** PERMANOVA statistical results for Bray–Curtis, unweighted UniFrac and weighted UniFrac distances analysed by environment type, condition (captive or free-living) and sample type (intestinal or faecal). All *p*-values are Holm corrected.

variable	Bray–Curtis			unweighted UniFrac			weighted UniFrac		
	*p*-value	*F*	*R*^2^	*p*-value	*F*	*R*^2^	*p*-value	*F*	*R*^2^
environment	0.012	12.87	0.29	0.012	12.39	0.28	0.012	10.77	0.25
condition	0.012	19.55	0.17	0.012	18.73	0.16	0.012	14.06	0.13
sample type	0.012	13.39	0.12	0.012	11.67	0.11	0.012	7.81	0.07

### (b) DNA extraction, 16S rRNA gene amplification and sequencing

DNA was extracted from all samples using the E.Z.N.A. Stool DNA Kit (Omega Bio-Tek; Norcross, GA) according to the manufacturer's instructions, except that DNA was eluted into 50 µl of elution buffer. In addition to all biological samples, we also processed one positive and six negative extraction controls.

Amplification of the V4 region of the 16S rRNA gene was performed through Nextera preparation methods, following the Amplicon PCR workflow in the Illumina 16S Metagenomic Sequencing Library Preparation Guide [15]. We performed paired-end 250 bp read sequencing on a MiSeq500 at the University of California Davis DNA Technologies core. Samples were sequenced in two separate library runs; all populations except the UN2 samples were sequenced on a single MiSeq500 lane, while the UN2 samples were sequenced on a separate run. In both sequencing runs we included a positive control (ZymoBIOMICS Microbial Community DNA Standard; Zymo Research; Irvine, CA) and confirmed fidelity of its composition. There were run effects when comparing between *Peromyscus* samples (*p* = 0.001, PERMANOVA); however, we believe these differences primarily reflect biological variation between populations rather than artefacts of sequencing run differences given that individuals from the same environment type showed highly similar microbial composition (see Results).

### (c) Quantitative PCR

Quantification of bacterial concentration was performed via quantitative PCR (qPCR) on a Bio-Rad CFX-96 machine (Bio-Rad; Hercules, CA) and analysed using the CFX Maestro software. Extracted DNA was PCR amplified using 515f/806r primers and Power SYBR Green PCR master mix (Applied Biosystems; Waltham, MA) using a standard protocol [16]. Bacterial load (concentration of bacterial cells per gram of faeces) was calculated based on a standard curve created from the DNA extraction product of a known quantity of bacteria from the ZymoBIOMICS Microbial Community Standard, adjusting for weight of faecal material extracted.

### (d) Data processing

Sequence data were demultiplexed and fastq sequences were then trimmed through cutadapt (version 3.4 with Python 3.9.5, [17]). Using the dada2 R package (version 1.16.0, [18]), sequences were filtered based on quality sequence scores, dereplicated, and forward and reverse amplicon pairs were merged. Taxonomy was assigned to amplicon sequence variants (ASVs) according to the SILVA database version 138.1 [19], and contaminants identified from the extraction and PCR negative controls were removed using the decontam R package (version 1.10.0, [20]). ASVs which mapped to chloroplasts, mitochondria and non-bacteria, or those that were not mapped at the phylum level were also removed. Of the 10 583 ASVs initially identified, 8064 were retained.

The median read count for biological samples was 91 169 (±36 925 (s.d.), range 19 962–226 848). Samples were alpha rarefied using the R package GUnifrac (version 1.3, [21]) to a depth of 19 900 reads. Raw sequencing data are available through ENA (accession no. PRJEB51510).

### (e) Statistical analysis

All statistical analyses were non-parametric to account for non-normal distributions and were carried out in R (version 4.2.0, [22]). Beta-diversity metrics and ordinations were calculated using the phyloseq package (version 1.36.0, [23]). Non-metric multi-dimensional scaling (NMDS) stress values are reported after 20 tries whether or not convergence was reached. Convergent solutions were found for weighted and unweighted UniFrac but not Bray–Curtis. Permutational MANOVA (PERMANOVA) was carried out with the adonis2 function in the package vegan (version 2.5-7, [24]). Alpha-diversity indices (Shannon index, ASV richness) were calculated on the rarefied ASV table and compared between groups using Kruskal–Wallis tests with a Holm multiple hypothesis correction. Variability in microbial community composition between environment types was calculated with the betadisper and permutest functions in vegan. To test for correlation between geographical and beta-diversity distances, a Mantel test was performed using the mantel function in vegan. This test was performed with a single randomly selected sample from each population in order to control for zero distances within populations. Bacterial load was compared between groups using Kruskal–Wallis tests with Holm multiple hypothesis correction. Differential abundance of microbial taxa between groups was calculated using analysis of composition of microbiomes with bias correction (ANCOM-BC) using the ANCOMBC package (version 1.2.2, [25]).

## 3. Results

Overall gut composition in all populations was dominated by Firmicutes (57.10% mean ±7.78 s.d.) and Bacteroidetes (34.15 ± 8.55) as is typical in the rodent gut (e.g. [26]). However, there were only minimal levels (1.44 ± 1.99) of Proteobacteria.

**Figure 1.**
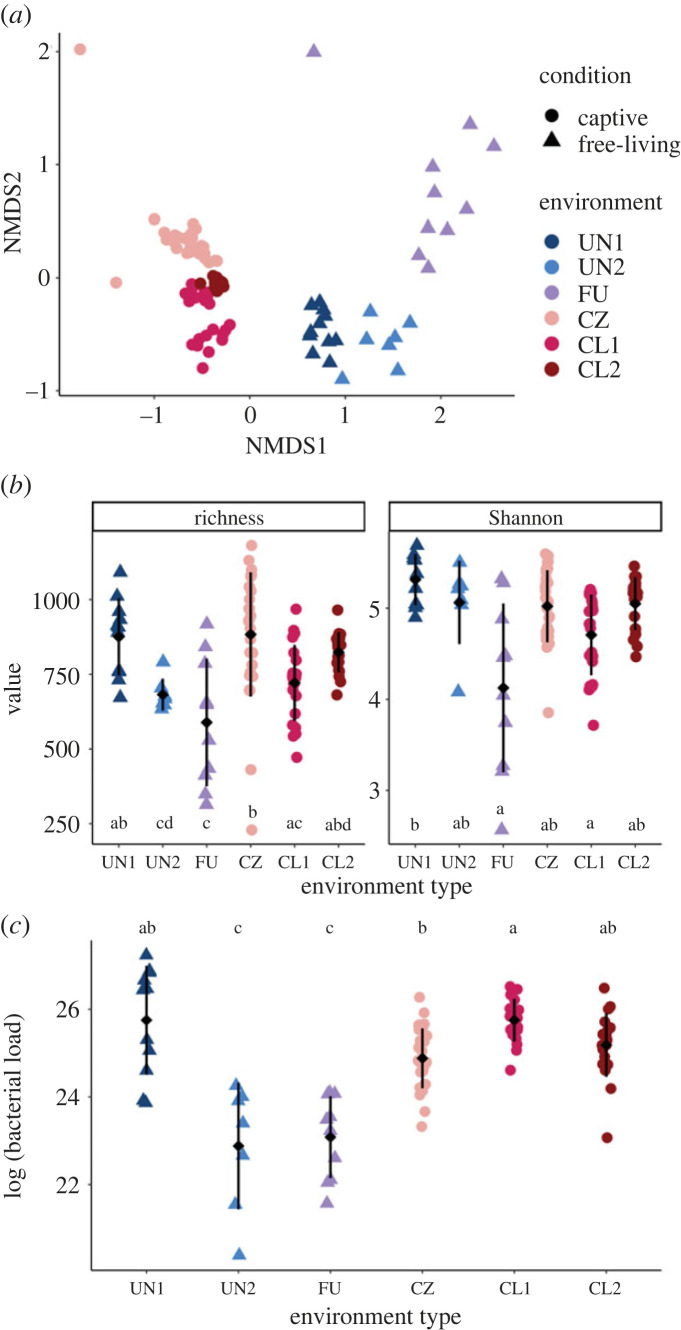
(*a*) NMDS ordination based on Bray–Curtis dissimilarity showing samples by environment type (colours) and condition (shape). NMDS stress = 0.09. (*b*) Alpha-diversity metrics richness and Shannon index by environment type. (*c*) Bacterial load (expressed as cells per gram of faeces) by environment type. Error bars in (*b*,*c*) indicate 1 s.d. from the mean. Groups not sharing any letter are significantly different based on a Holm-adjusted Dunn test and a significance level of 0.05.

We observed significant variation in gut microbial composition between environment types and between captive versus free-living conditions, with environment type explaining the greatest proportion of the variation (figure 1*a*, table 2). The results for environment type (*p* = 0.012, PERMANOVA) and condition (*p* = 0.012) were statistically significant and were robust to dissimilarity metric used (for weighted and unweighted UniFrac results see table 2; electronic supplementary material, figure S2). Within the free-living populations, urban and undeveloped composition were significantly different (*p* = 0.001). In addition, we found sample dispersion was significantly different between environment type (*p* = 0.003, permutation test, Holm corrected), condition (*p* = 0.003) and sample type (*p* = 0.003). Geographical distance between populations did not correlate with Bray–Curtis dissimilarity (*p* = 0.82, Mantel test; electronic supplementary material, figure S3).

The diversity metrics Shannon index and ASV richness both varied significantly between environment types (*p* < 0.005, Kruskal–Wallis tests), but neither showed significant differences when analysed by condition or sample type (*p* > 0.1). The free-living urban population had the lowest mean bacterial diversity and richness and had significantly lower richness than a least one population for each of the other environment types (*p* < 0.05, Holm-adjusted Dunn tests). Bacterial diversity and richness of deer mice from undeveloped environments did not differ from that of the captive deer mice (*p* > 0.1, figure 1*b*). Bacterial load also significantly differed between environment types (*p* < 0.001, Kruskal–Wallis test, figure 1*c*) and housing conditions (*p* < 0.001) but not sample type (*p* = 0.1). Two populations, the urban and an undeveloped, had significantly lower bacterial load than all other populations (*p* < 0.05, Holm-adjusted Dunn tests).

**Figure 2.**
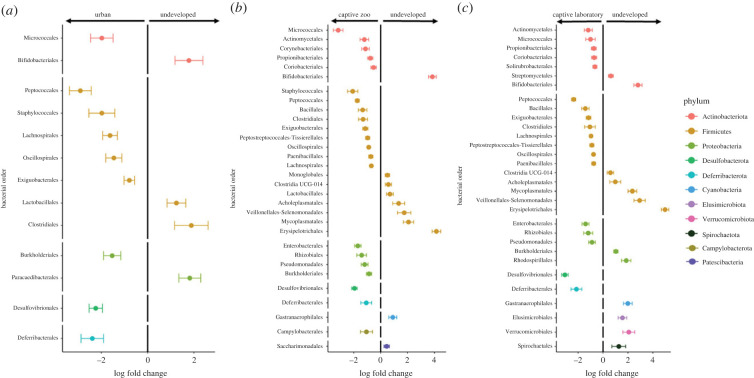
ANCOM-BC results between developed and undeveloped environments. Positive log fold change indicates taxa enriched in undeveloped environments, while negative log fold change indicates taxa enriched in (*a*) urban, (*b*) captive zoo and (*c*) captive laboratory environments. Bacterial orders are shown clustered by phylum. Error bars indicate 1 s.d. from the mean.

To identify specific taxa associated with housing condition, we ran differential abundance testing with bias correction (ANCOM-BC) at the order level. We compared undeveloped environments to urban, captive zoo and captive laboratory environments in a pairwise manner (figure 2). In all three comparisons, deer mice from undeveloped environments had enriched Bifidobacteriales and reduced levels of Deferribacterales, Desulfovibrionales, Micrococcales and four orders of Firmicutes. Of the nine orders enriched in urban deer mice, all were also enriched in at least one captive environment (electronic supplementary material, figure S4).

## 4. Discussion

Laboratory, zoo and urban environments are all places where animals can experience altered diets, stress and exposure to bacteria from humans and built environments—factors that are expected to impact their gut microbiome composition. Despite these similarities, the gut microbiota of captive deer mice in our study did not resemble those of urban deer mice, while captive zoo and laboratory populations were fairly similar. Although we observed patterns of enrichment of a few bacterial orders in all developed environments, ultimately each environment type uniquely shaped the bacteria present.

Our findings substantiate and expand those of the only other study that compared the gut microbiota of deer mice in wild and captive settings [14]. Schmidt *et al*. similarly found wild and captive bred deer mice differed in microbiome composition, including an enrichment in Enterobacterales as we observed. However, our findings disagree on the effect of captivity on gut microbial diversity and the abundance of other taxa such as Deferribacterales and Desulfovibrionales. We found distinctive microbial characteristics at the population level indicating local ecological characteristics also matter for shaping wild and captive microbiomes. This fine-scale variation likely contributes to the general finding, shown across the literature of many host species, of inconsistent effects of captivity both in terms of enriched taxa and whether diversity increases or decreases (reviewed in [27]).

Focusing on captive populations, we found that samples from laboratory and zoo deer mice were largely similar in gut microbial composition and diversity. To our knowledge, this has not been shown in any other species. The three captive settings we sampled differed in the housing, diet and medical care provided as well as in how long the deer mice had been subject to captivity and their original source population. Despite experiencing limited handling and a more natural diet, the microbiota of the captive zoo population was much more similar to those of laboratory populations than that of the urban population found on the grounds of the same zoo. This suggests that attempts to emulate the natural environment in captivity may not be sufficient to prevent a shift to a captive microbiota, consistent with another recent study [28]. The smaller differences found between the microbiota of captive populations parallel findings that laboratory mice from different vendors or at different facilities also have distinct microbiota [29].

By contrast, urban deer mice had quite different gut microbiota compared to those from captive environments. Urban mice typically showed higher beta-dispersion and lower bacterial load, as well as generally lower diversity, compared to the other environments (with the exception of one undeveloped population which also had low bacterial load). However, while their gut composition was dissimilar at the ASV level, they did share many enriched orders with the captive mice, compared to undeveloped mice. No other study has previously compared the gut microbiome of urban deer mice to other mice. Work in other animals generally finds undeveloped and urban animals differ in their microbiome composition, with greater diversity in the urban populations [4], but again data are lacking for comparisons of urban and captive animals.

The distinct urban deer mice and similarity between captive populations lead to the result that environment type superseded any geographical effect on the gut microbiota. Geographical location has previously been shown to play a large role in shaping mammalian gut microbial composition [2,30], sometimes to the extent that it overcomes signals of environment type, as in a recent study of captive and wild lemurs [8]. The similarity of the two populations from undeveloped locations, located several states away, confirms the limited effect of geography in our study.

Other factors known to impact the gut microbiome (e.g. [2,31]) are beyond the scope of our analyses due to limitations of our study and gaps in sampling metadata. Subspecies sometimes [32], but not always [33], can influence the mouse gut microbiota. We observed only minimal differences between two subspecies housed at CL1 but lack complete subspecies data thus precluding a comprehensive analysis. We did not have sufficient data to test for sex effects (although where sex was known, populations were evenly distributed), but sex typically explains a minor component of microbiome variation in rodents [34]. By contrast, season is well-known to shape the microbiome, likely largely due to seasonal diet fluctuations [35]. We do not expect that seasonality played a role in shaping these results, as most samples were collected during the summer. While samples from the two captive laboratory populations were collected in winter, these deer mice receive a constant diet year-round which should limit seasonal fluctuations.

The urban population we sampled may potentially be considered idiosyncratic, thus limiting our ability to draw broader inferences. While the zoo is located within a highly industrialized major US city and all individuals were trapped in human-occupied buildings, the environmental exposures on zoo grounds may not be representative of a typical city environment. It is unknown, for instance, whether the deer mice had access to animal enclosures. No other studies have published gut microbiome sequences for urban deer mice, so we cannot assess how representative our findings are.

There are several potential explanations for the distinct nature of urban microbiota, such as their diet combining natural and anthropogenic food sources, exposure to both indoor and outdoor environmental bacteria, and stress associated with the urban environment [36]. At this point, we cannot disentangle the potential effects of these factors. Both diet and stress have been shown to impact gut microbial composition in urban and captive settings [36,37]; however, it is unknown how and to what extent these impact the deer mouse system.

While we cannot pinpoint specific reasons for the composition and diversity patterns observed here, the result that the microbiota of urban animals do not resemble those of captive animals provides a valuable starting point for future studies investigating anthropogenic impacts on wild animals and their microbes. Moreover, our results accentuate the limitations of laboratory animals and newly highlight the limits of zoo animals as models for studies of the microbiome. Caution should be taken when interpreting results of microbiome studies beyond captive settings, even to other anthropogenic environments.

## 5. Conclusion

Our analysis provides the first comparison of how several different developed environments influence the gut microbiota of a single species of rodent. We show that the gut microbiota of deer mice from captive, urban and undeveloped environments are distinct. The factors shaping microbial composition in developed environments are complex, and similarities between environments such as geographical proximity or exposure to humans may not confer a particular ‘developed’ microbial community.

## Data Availability

Raw sequencing data are available through ENA (accession no. PRJEB51510).
